# The Profiles of Low Molecular Nitrogen Compounds and Fatty Acids in Wort and Beer Obtained with the Addition of Quinoa (*Chenopodium quinoa* Willd.), Amaranth (*Amaranthus cruentus* L.) or Maltose Syrup

**DOI:** 10.3390/foods9111626

**Published:** 2020-11-07

**Authors:** Paulina Bogdan, Edyta Kordialik-Bogacka, Agata Czyżowska, Joanna Oracz, Dorota Żyżelewicz

**Affiliations:** 1 Institute of Fermentation Technology and Microbiology, Faculty of Biotechnology and Food Sciences, Lodz University of Technology, 171/173 Wolczanska Street, 90-924 Lodz, Poland; edyta.kordialik-bogacka@p.lodz.pl (E.K.-B.); agata.czyzowska@p.lodz.pl (A.C.); 2 Institute of Chemical Food Technology, Faculty of Biotechnology and Food Sciences, Lodz University of Technology, 9/10 Stefanowskiego Street, 90-924 Lodz, Poland; joanna.oracz@p.lodz.pl (J.O.); dorota.zyzelewicz@p.lodz.pl (D.Ż.)

**Keywords:** beer, yeasts, *Saccharomyces pastorianus*, unmalted raw materials, quinoa, amaranth, fatty acids, amino acids, esters, higher alcohols

## Abstract

Replacement of a part of malt with unmalted materials is a common practice in beer production. These materials may differ in chemical composition than barley malt, which in turn can contribute to changes in the final composition of the wort. Consequently, it may affect yeast metabolism and final parameters of the obtained products. In this research, two unmalted pseudocereals were used: quinoa *(Chenopodium quinoa* Willd.) and amaranth (*Amaranthus cruentus* L.). Maltose syrup was tested as a reference material due to its commercial usage as a substitute of malt in production of worts. Replacement of a part of the malt with quinoa or amaranth favorably influenced the profiles of amino and fatty acids. Due to the fact that the type and concentration of individual amino acids and fatty acids in the fermented wort significantly affect the flavor compounds synthesized by yeast, differences in the profiles of esters and higher alcohol have been noted in beers produced with pseudocereals.

## 1. Introduction

Wort is an intermediate in the production of beer, obtained by mashing malt with water and then boiled with hops. The mashing process involves a transfer of partially or completely hydrolyzed compounds from malt and/or unmalted raw materials to water, which allows obtaining wort rich in nutrients necessary for the yeast’s metabolism [1]. The optimal concentration of fatty acids (FA) and free amino nitrogen (FAN) in fermented wort and beer are being considered as crucial for proliferation of yeasts as well as final taste and aroma of beer [2]. It is believed that higher concentration of fatty acids can negatively affect the quality of beer. Although the content of long-chain fatty acids is usually very low in the final product, an increase in their concentration, together with inadequate storage conditions, may lead to an increase in the content of compounds formed through their oxidation, which affects beer’s qualities [3]. On the other hand, fatty acids are essential for the growth of yeast cells and can significantly affect the yield of fermentation [4]. What is more, the other group of compounds-free amino nitrogen (FAN) (amino acids, ammonium ion, peptides, and tripeptides) are utilized by yeasts in the formation of a new cells [5]. In addition, amino acids are converted by yeasts to higher alcohols, from which esters are synthesized [2]. Consequently, the profile and content of individual amino acids in the wort significantly affect the concentration of compounds responsible for beer’s flavor bouquet. Finally, the content of FAN in wort obtained with the use of different raw materials is one of the most important quality indicators [6].

The substitution of malt with unmalted raw materials is a common practice in brewing [7]. Due to several advantages, such as reduction of production costs, easy dosing, an increase of the extract content in High Gravity Brewing technology, and lightening of the color or reduction of viscosity, maltose syrups are often used as a substitute for barley malt [7]. On the other hand, their use also could bring some risks. Except sugars, starch syrups do not contain compounds necessary for yeasts such as nitrogen compounds, fatty acids, or micro- and macroelements. Consequently, it can adversely affect the fermentation process as well as the viability and the vitality of the yeasts [8]. Therefore, new malted and unmalted raw materials are still sought after. One of the requirements of such materials is the content of ingredients necessary for proper propagation of yeasts and following effective fermentation. As a result, pseudocereals like quinoa (*Chenopodium quinoa* Willd.) and amaranth (*Amaranthus cruentus* L.) arouse more and more interest.

Since quinoa and amaranth are richer in nutrients than traditional cereals, growing interest in these plants among scientists, dieticians, and consumers is observed [9]. Their seeds are characterized by a high content of starch (50–69%) and minerals such as zinc, magnesium, potassium, calcium, phosphorus, as well as vitamins: riboflavin, thiamine, and vitamins B_6_ and E [10]. In addition, they contain a large amount of proteins (12–23%), characterized by a rich composition of essential amino acids [9]. In contrast to traditional cereals, these pseudocereals do not contain prolamins, which are unfavorable for people with celiac disease [11]. What is more, fat contents in seeds of quinoa (5.2–9.7%) and amaranth (5.7–10.9%) are higher than in the case of barley (1.5–2%) [12]. The ratio of unsaturated fatty acids to saturated fatty acids is higher in quinoa than in amaranth [11]. Furthermore, fatty acids found in quinoa and amaranth are generally not susceptible to oxidation. This is related to the high content of antioxidants: tocopherol (vitamin E) and tocotrienol (a precursor of vitamin E) as well as carotenes [13]. Therefore, they may be valuable to yeast without adversely affecting the flavor of the beer during storage.

Previous studies have shown that the sensory evaluation of beer obtained in the production with quinoa and amaranth received higher scores compared to the malt beer [14]. However, so far, no studies have been carried out showing whether quinoa and amaranth affect the composition of the wort with ingredients that can improve the effectiveness of the fermentation process. Therefore, this work aims to show how the partial replacement of malt with quinoa, amaranth, or maltose syrup changes the content of fatty acids and amino acids in wort, which may affect the efficiency of the fermentation process and the synthesis of fermentation byproducts.

## 2. Materials and Methods

### 2.1. Raw Materials

Quinoa (*Chenopodium quinoa* Willd.) seeds were obtained from the ecological crops of Bolivia while amaranth (*Amaranthus cruentus* L.) seeds were obtained from the ecological crops of India (distributor Bio Planet, Wrocław, Poland). Flakes were obtained from seeds of both species (distributor Bio Planet, Wrocław, Poland). Pilsen malt was obtained in 2015 by local commercial manufacturer (Optima, Poznań, Poland). Maltose syrup with a low content of glucose (up to 8%) and high content of maltose (45–54%) was provided by local distributor (Cargill, Warsaw, Poland). Worts were boiled with 1.5 g/L hop pellets (Marynka type 45) (Powisle, Wilków, Poland) containing 8.7% of α-acid.

### 2.2. Yeasts

Bottom fermentation yeasts *Saccharomyces pastorianus* W34/70 were used in the fermentation. The strain was purchased from the pure culture collection of yeasts Hefebank Weihenstephan (Au i. d. Hallertau, Germany). To evaluate the purity of the obtained strain, the biomass was streaked onto YPG agar medium (Merck, Darmstadt, Germany) and incubated at 25 °C ± 2 °C for 72 h. Afterwards, single colonies were transferred onto the YPG slants, incubated in analogous conditions, and then maintained as pure cultures at 4 °C until usage. The activation of the yeasts before the fermentation was conducted in 200 mL of YGP broth. The medium was inoculated with a single colony of the pure culture, and incubated for 24 h at 25 °C ± 2 °C. The culture was used in the inoculation of the worts with 1.2 × 10^7^ cells/mL (using the Thoma chamber).

### 2.3. The Production of Worts and Beers

Pilsen malt, quinoa, and amaranth flakes were ground using laboratory disc mill DLFU (Bühler Group, Uzwil, Switzerland). The settings were chosen so that the final particles diameters of the particles were 0.1 mm for quinoa and amaranth, and 0.2 mm for malt. Subsequently, mashing with various variants was carried out: (1) barley malt (control); (2) 70% of barley malt with 30% of quinoa; (3) 70% of barley malt with 30% of amaranth; (4) 70% of barley with 30% of maltose syrup. Dry mass of tested variant (50 g) was mixed with 200 mL of water and mashed using Mash Bath R-12 (1-Cube, Havlíčkův Brod, Czech Republic) as follows: 30 min at 45 °C, 60 min at 62 °C, 30 min at 72 °C, and 10 min at 78 °C (mashing-off). Mashing temperature increased by 1 °C per minute, with continuous mixing. 

**Figure 1:**
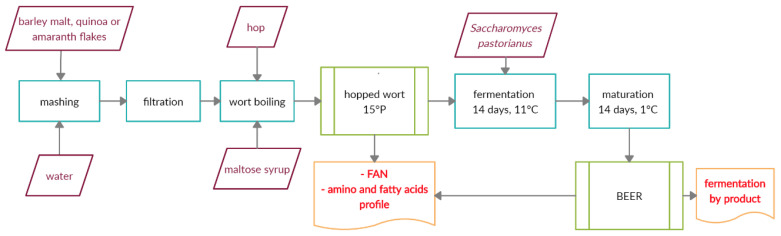
Scheme of wort and beer production with the conducted analyses during the research.

After that, the hot mashes (76–77 °C) were filtered using MN 614 1/4 filter paper (Macherey-Nagel, Düren, Germany) (Figure 1). Then, worts were boiled for 60 min with hop pellets (1.5 g/L). Half of the hop pellets were added at the beginning of the boiling, while the remaining half were added 10 min before the end of the process. In the case of beers produced with the addition of 30% of maltose syrup, this raw material was added after the boiling. The worts were then filtered using MN 614 ¼ filter paper (Macherey-Nagel, Düren, Germany) to remove the hot break. After separation of the hot break, the extract from each wort was adjusted to 15 ± 0.2 °P using sterile tap water. Following this, the physicochemical analyses were carried out for the obtained worts (free amino nitrogen and amino and fatty acid profile). After the inoculation with yeasts, fermentation was carried out at 10 °C for 14 days. After fermentation, beers were transferred to glass bottles and matured for 14 days at 1 °C. Then, beers were clarified by adding diatomaceous earth (50 g/L), filtered using MN 614 ¼ filter paper (Macherey-Nagel, Düren, Germany) and analyzed. The production of worts and beers was carried out in triplicates.

### 2.4. The Fatty Acids Determination in Worts and Beers

The fatty acid profile of wort and beer was determined by Gas Chromatography—Flame Ionization Detector (GC/FID) after saponification with methanolic NaOH solution (2%) followed by transesterification with 14% methanolic solution of BF3 [15]. Methylated fatty acids were then analyzed using Varian 450 gas chromatograph equipped with FID detector (Viarian, Inc., Palo Alto, CA, USA). Fatty acids separation was performed on a FactorFour VF-23ms column (60 m × 0.25 mm × 0.25 mm) (Viarian, Inc., Palo Alto, CA, USA). For separation, the temperature range of 180–220 °C for 25 min was used with 28 °C/min rate and 5 min heating of the column at 220 °C between the experiments. The temperature of injector was 220 °C and the detector temperature equaled 250 °C. Helium was used as a carrier gas with a flow rate of 1 mL/min and 1:100 split. Standards of fatty acids were purchased from Sigma-Aldrich (Milan, Italy).

### 2.5. Determination of Low Molecular Nitrogen Compounds and Amino Acids

The Free Amino Nitrogen (FAN) in hopped worts and beers were analyzed with accordance to the standard methods described in Analytica EBC Method 8.10 (wort) and EBC Method 9.10 (beer). The determination of free amino acids was performed with accordance to the method described by Dudkiewicz et al. (2016) [16]. Briefly, worts and beer were passed through a centrifugal ultrafilter with a cut-off mass of 3 kDa (Amicon^®^ Ultra Centrifugal Filters, Merck Millipore, Burlington, VT, USA). Then, 50 µL of resulting samples were then transferred into Eppendorf tubes and evaporated to dryness in a vacuum centrifuge. The free amino acids present in the dried sediment had been converted into a phenylthiocarbamide (PTC) derivatives. The obtained derivatives were dissolved in amino acid solvent (PigoTag diluent WAT088119), and 5 µL solutions were analyzed using high pressure liquid chromatography (HPLC) on a PicoTag 3.9 × 150 mm column (Waters Corporation, Milford, MA, USA). Quantitative calibration had been performed using four different concentrations of Pierce™ Amino Acid Standard (Thermo Fisher Scientific, Waltham, MA, USA).

### 2.6. Determination of Fermentation by Products

Chromatographic analysis of the volatile compounds (esters and higher alcohols) in beers was carried out using a GC apparatus (Agilent 7890A, Santa Clara, CA, USA) with a mass spectrometer (Agilent MSD 5975C, Santa Clara, CA, USA), according to the method described by Balcerek and Pielech-Przybylska (2016) [17].

### 2.7. Statistics

Means with standard deviations (±SD) were calculated from the data obtained from three independent experiments. The values were compared using one-way repeated measures analysis of variance with Tukey test using STATISTICA 9.0 software (TIBCO Software Inc., Palo Alto, CA, USA).

## 3. Results and Discussion

### 3.1. Fatty Acid Profiles in Worts

Most of the lipids in the wort come from malt or unmalted raw materials [18]. In this study 14 fatty acids, including 5 saturated fatty acids were detected by gas chromatography. The highest content of fatty acids was determined in the wort obtained with 30% of amaranth (5.86 mg/L) and quinoa (5.51 mg/L) (Table 1). As a result of the replacement of 30% of malt with amaranth during the production of wort, the concentration of fatty acids increased by 25%, compared to barley wort (4.40 mg/L). On the other hand, the replacement of 30% of malt with quinoa resulted in an increase in the content of determined fatty acids by 19%. The obtained results were in line with the previous reports, which indicated that the fat content in quinoa or amaranth is at least twice higher compared to barley and barley malt [10,14].

Potential advantages of the use of lipid-rich worts and their positive effect on the fermentation are debatable. Some reports do not recommend the use of raw materials rich in fats in the production of worts, because high concentration of these compounds can negatively affect the organoleptic qualities and stability of beers [19]. On the other hand, in worts with a high content of fatty acids, a better proliferation of brewer’s yeasts, their greater viability and shorter time of fermentation were observed [20,21,22]. Stewart and Martin (2004) [23] reported that during the fermentation in the medium with higher content of fats, yeasts were characterized by increased use of FAN, while Schisler et al. (1982) [21] found more efficient synthesis of ethyl alcohol.

From another point of view, Moonjai et al. (2003) and Hull (2008) observed that a higher content of fatty acids in the wort causes a decrease in the oxygen demand of brewer’s yeasts [24,25]. When fatty acids are present in a higher concentration, synthesis of the compounds by yeasts is not required, and thus, the demand for oxygen is lower [26]. Consequently, less oxygenation can reduce oxidative stress and contribute to better flavor stability of the beer. The increased fatty acid content is particularly important in the fermentation of worts with a high extract, in which oxygen solubility is limited [19]. Thus, the use of quinoa or amaranth as partial substitutes for barley malt in worts production may reduce the need for increased oxygenation.

The total values of the analyzed fatty acids indicated that the replacement of the barley with pseudocereals increases the concentration of FA to 5.86 ± 0.01 mg/L (amaranth) and 5.51 ± 0.02 mg//L (quinoa). The highest values were noted for oleic (1.46 mg/L and 1.34 mg/L), stearic (0.81 mg/L and 0.75 mg/L), palmitic (0.77 mg/L and 0.71 mg/L), α-linolenic (0.55 mg/L and 0.45 mg/L), and linoleic acids (0.30 mg/L and 0.37 mg/L), respectively for amaranth and quinoa. On the other hand, the concentration of myristic acid was lower in the samples with quinoa and amaranth, and in both cases, it was 0.15 mg/L. It is worth to note that linoleic acid is known for having a significant impact on fermentation efficiency and beer quality. It has been observed that in wort with increased content of linoleic acid, yeasts proliferate more effectively and show greater viability. In consequence, the fermentation process is faster, and the synthesis of ethyl alcohol is more efficient, without increased content of higher alcohols [24,26]. Thurstona et al. (1982) found a direct correlation between the level of linoleic acid from malt and the level of ester synthesis during fermentation [27]. It appears that a higher concentration of linoleic acid can lead to a less efficient synthesis of esters, particularly of ethyl acetate and isoamyl [28]. Our previous research also showed a smaller amount of yeast-synthesized esters in beers obtained with quinoa (7.89 mg/L) or amaranth (6.93 mg/L) in comparison to barley beer (8.82 mg/L) [14]. Furthermore, as an effect of the addition of oleic acid (18:1), decreased concentration of synthesized esters can be observed [19]. Kalmokoff and Ingledew (1985) showed that during the fermentation of wort supplemented with oleic acid (9.1 mL/L), the rate of assimilation of low molecular nitrogen was two-times higher, while the synthesis of ethyl alcohol was up to 50% more efficient [28]. These indicate that the supplementation of wort with quinoa or amaranth can cause an increased content of essential fatty acids, which in turn can contribute to faster yeasts proliferation, as well as to the greater absorption of low molecular nitrogen compounds, including amino acids.

### 3.2. Fatty Acid Profiles in Beers

During the fermentation, some of the fatty acids were assimilated by the yeasts. The highest content of fatty acids was determined in beer obtained with 30% of amaranth. In comparison to malt beer (2.04 mg/L), the content of these compounds was 22% higher in beer with 30% of amaranth (2.49 mg/L), and 9% higher for 30% of quinoa (2.23 mg/L). During the fermentation of the wort with a 30% of maltose syrup, yeast assimilated only 44% of fatty acids, while the assimilations from wort obtained with a 30% of amaranth or wort obtained with a 30% quinoa were on the level of 57% and 60%, respectively (Table 1). This shows that the activity of yeasts during the fermentation of worts obtained with the use of pseudocereals is much more efficient than during the fermentation of wort with a 30% of maltose syrup. What is more, during the fermentation of wort obtained with the quinoa, oleic, α—linolenic, γ—linolenic, palmitic, and linoleic acids were the most used (over 60%) by the yeasts. Apart from these fatty acids, heptadecanoic acid has also been utilized in a significant part from the wort obtained with the use of amaranth. In the case of barley wort, yeasts used mainly oleic, linoleic, and α-linolenic acids, while from the wort obtained with the addition of maltose syrup, α-linolenic, oleic, and palmitic acids were used. Despite the differences in the assimilation of individual acids by yeasts fermenting various types of worts, the general tendency was the very high rate of use of α-linolenic and oleic acids.

During fermentation of all worts, unsaturated fatty acids were greatly utilized by yeasts, compared to the assimilation of saturated fatty acids. During the fermentation of the wort obtained only from barley malt, yeast assimilated 75% of unsaturated fatty acids and 38% of saturated fatty acids. The utilization of unsaturated fatty acids was more notable in the case of quinoa and amaranth, respectively, 80% and 78% of utilization, while in the case of saturated fatty acids, these values were respectively 42% and 40%. The lowest degree of utilization of both saturated and unsaturated fatty acids was noted in the fermentation of wort obtained with 30% of maltose syrup (32% and 64%, respectively). Therefore, it is clearly visible that regardless of the fermentation medium, yeast is more likely to assimilate unsaturated fatty acids.

Yeasts metabolism is associated with the formation and release of short-chain fatty acids (C6:0 to C10:0) [29]. As a result, the low molecular weight fatty acids present in beer do not come exclusively from wort but are synthesized by yeasts cells during multiplication and fermentation. The concentration of short-chain fatty acids synthesized by yeasts may indicate their physiology. The highest concentration of short-chain fatty acids (C6:0 to C10:0) was noted for beer obtained with a 30% of amaranth (0.69 mg/L) or quinoa (0.63 mg/L), while the lowest concentration was observed in beer obtained with a 30% of maltose syrup (0.42 mg/L). Thus, the growth and activity of yeasts during the fermentation of wort obtained with a 30% of pseudocereals is higher, especially in comparison to wort produced with the addition of maltose syrup.

### 3.3. The Content of Free Amino Nitrogen in Worts and Beers Supplemented with Pseudocereals

Unmalted raw materials, even those with a higher content of proteins than in barley malt, usually cause a decrease in FAN content in wort. It is associated with a lower content of endogenous enzymes and more compact structure of endosperm. In turn, starch syrups contain mostly sugars [7]. The highest content of FAN was determined in the wort obtained from barley malt (Table 2). Worts obtained with 30% addition of amaranth (208.4 mg/L) and quinoa (198.9 mg/L) were 13% and 17% lower thanthat of the barley malt. Lower content of FAN in the wort obtained with maltose syrup (134.13 mg/L), with a high extract content (15 °P may contribute to the slower yeasts proliferation, which results from the fact that these compounds are necessary inter alia for the formation of cell walls and fermentation [30].

In addition to differences in FAN profiles, clear differences in the assimilation of low-molecular nitrogen compounds were also noted (Table 1). The majority of FAN were used by yeasts from wort obtained with 30% of quinoa (77%). Wort with a 30% of maltose syrup was characterized by the lowest content of nitrogen compounds, the rate of FAN assimilation was the lowest (46%). Probably, the qualitative composition of the nitrogen compounds of the wort obtained with 30% quinoa was more favorable for yeasts. The substitution of 30% of malt with amaranth flakes, resulted in increased assimilation of FAN. However, the assimilation was lower than that obtained with the addition of quinoa. Increased use of FAN by yeasts, as a result of the substitution of a part of malt with unmalted oats, was also observed in the research conducted by Kordialik-Bogacka et al. (2014) as well as Aastrup (2010) [31,32].

### 3.4. The Amino Acids Profile in Wort Obtained with Addition of Adjuncts

To confirm the presence of more favorable amino acids in worts obtained with the participation of pseudocereals, the profiles of selected amino acids in worts were determined by HPLC (Table 3). Worts usually contain up to 20 amino acids, which are used by yeasts in a specific order: lysine, arginine, asparagine, aspartate, glutamate, serine, and threonine (group A); histidine, leucine, isoleucine, methionine, and valine (group B); and alanine, glycine, phenylalanine, tyrosine, and tryptophan (group C). Proline is classified in a separate group D [30]. In our study, the content of 6 amino acids from group A, 5 representatives from group B, and 4 from group C was determined. Additionally, proline was identified. The use of unmalted raw materials affected not only the differences in the total content of amino acids in worts but also the amount of individual amino acids, including those of great importance for yeasts activity (Table 3).

The results of our study indicate that the application of pseudocereals decreased the total content of amino acids in worts. In comparison to barley malt (1003.2 mg/L), the values were 18.6% and 16.8% lower, respectively, for amaranth (816 mg/L) and quinoa (834 mg/L) (Table 3). The application of maltose syrup resulted in a reduction in the quantity of all amino acids, proportionally to the percentage of maltose syrup in the wort. The lower results of the total amino acid content in pseudocereals were significantly influenced by the lower proline content in these materials. In amaranth, the content of proline was 205.4 mg/L, in quinoa 190.1 mg/L while for barley malt it was 290.2 mg/L. This is important due to the fact that this amino acid is considered as difficult to assimilate by brewer’s yeast, which in turn can significantly affect the beer production.

Thus, the use of pseudocereals may be advantageous for wort fermentation. In addition, a favorable change resulting from the use of amaranth and quinoa was noted in the case of lysine. Wort with 30% of amaranth showed increased content of lysine (40.8 mg/L) up to 19%, while in the case of 30% quinoa (44.6 mg/L), the value was 27% higher than in malt wort (32.9 mg/L). On the other hand, for malt syrup (19.7 mg/L), the value was lower by 40% than in the malt wort. According to Santos et al. (2014) [6] and Tenge (2009) [33] lysine is a key amino acid for yeasts, especially in the phase of their proliferation and the amino acid is completely used after several hours from the beginning of the fermentation. Lekkas and others (2007) [30] found that the supplementation with lysine causes faster proliferation of yeasts and a half shorter time of fermentation. On the other hand, Kolothumannil and Ingledew (1992) [34] indicated that wort should not be supplemented only with lysine, because this amino acid may cause growth inhibition and reduction of the viability, and worts should be enriched with various amino acids. The application of quinoa complies with these criteria. The content of glutamine in the wort obtained with a 30% of quinoa was 17% higher, while serine, threonine, and histidine were 16% higher. The content of methionine increased by 22%, isoleucine by 3%, and leucine by 11%, compared to malt wort. Thus, it can be stated that the addition of unmalted pseudocereals, such as quinoa and amaranth, significantly increases the amount of such important amino acid for yeasts. This is particularly important from the point of view of the worts fermentation rate and the possibility of shortening the time of this process, especially in the case when worts are characterized by a high extract content, because their fermentation needs more time and requires more biomass of yeasts.

**Figure 2:**
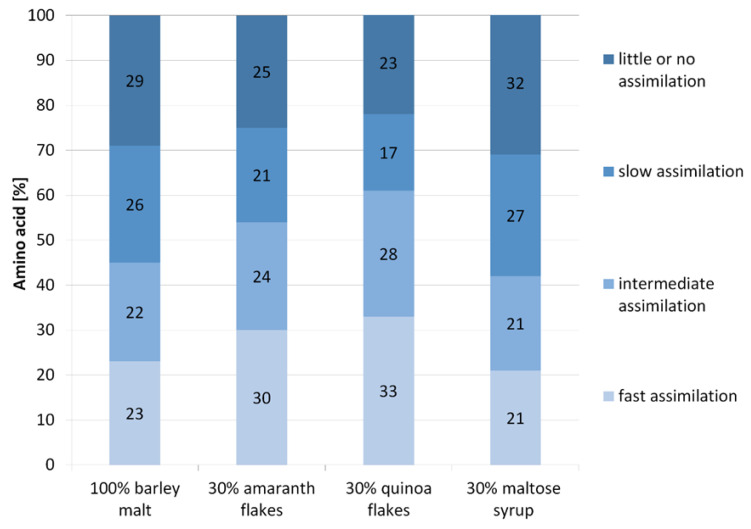
The percentage composition of the groups of amino acids in tested worts. Group A (fast assimilation), Group B (intermediate assimilation), Group C (slow assimilation), Group D (little or no assimilation).

However, changes in the content of groups of amino acids in worts obtained with the participation of various unmalted materials turned out to be the most interesting (Figure 2). The effect of substitution of 30% barley malt with pseudocereals was the increased content of amino acids from group A. The values increased from 23% to 30% and 33% of total amount of amino acids, respectively, for amaranth and quinoa. In turn, the addition of maltose syrup to the wort resulted in the least favorable amino acid profile; the content of the fast assimilable amino acids was the lowest and equaled 21%. At the same time, wort with maltose syrup was characterized by the highest values of group C (slow assimilable) and D (little or no assimilable) amino acids. In general, the most beneficial amino acids composition in terms of efficiency of fermentation and yeast propagation was determined in the wort obtained with 30% addition of quinoa—highest values for amino acids belonging to group A and lowest values for group D.

### 3.5. The Use of Amino Acids by Yeast during Fermentation

**Figure 3:**
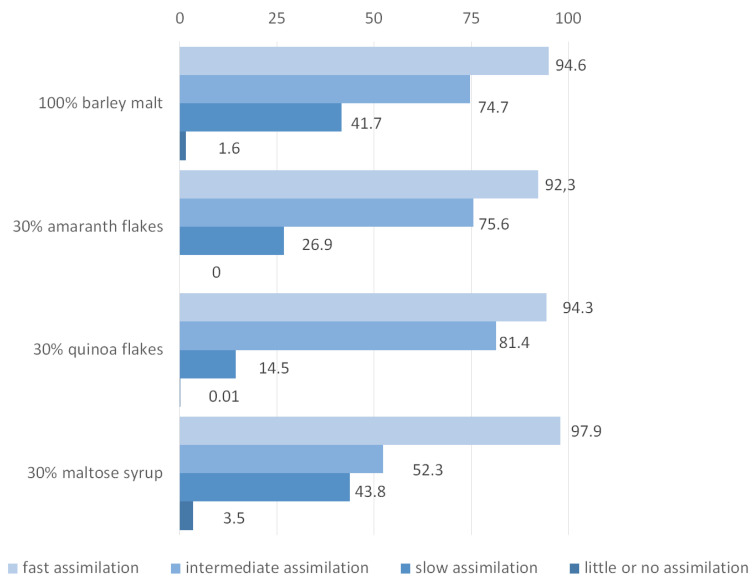
The assimilation [%] of the individual groups of amino acids by yeast during the fermentation, compared to the results obtained for amino acids in worts.

The highest degree of use of the amino acids during wort fermentation was found in beer obtained with 30% quinoa (56%), and the lowest in beer obtained with the addition of maltose syrup (44%) (Table 3). Analogous dependence was observed when all low molecular nitrogen compounds were considered (Table 2). In addition to the changes in the total amount of amino acids used by yeasts, the proportion between individual groups of assimilated amino acids has also changed (Figure 3). During the fermentation of the wort obtained with maltose syrup, the highest utilization of group A amino acids (97.9%) was observed. Amino acids from this group were used in a similar extent during fermentation of barley wort (94.6%) and wort obtained with 30% quinoa (94.3%). The lowest degree of assimilation was noted for the wort obtained with 30% of amaranth (92.3%), but the differences between the variants of fermented worts were not significant.

During the fermentation of wort obtained with 30% addition of quinoa, the most assimilated were amino acids from group A while the least from group C. Similar results were noted for the wort obtained from 30% amaranth. The most interesting was the assimilation of amino acids from individual groups during fermentation of wort with 30% maltose syrup. The shares of amino acids from groups B and C used by yeasts were similar and equalled 52.3% and 43.8%, respectively. Compared to other beers, the assimilation of group B amino acids was much weaker in this case. The highest degree of proline assimilation was recorded in beer obtained with 30% maltose syrup (3.5%) (Figure 3). There was no use of proline during fermentation of wort in the case in which 30% of the malt was replaced with quinoa or amaranth. The interpretation of the results is the answer to the question of whether the increased use of FAN by yeast in worts with the addition of quinoa is related to more favorable amino acid composition. Good assimilation of amino acids from groups A and B during the fermentation of the wort obtained with the addition of quinoa, together with the low utilization of slowly digestible amino acids with zero proline uptake, seems to be the most obvious from the point of view of currently available knowledge. Quinoa enriches the wort with amino acids, primarily from group A. The degree of utilization of amino acids from individual groups from the wort obtained with 30% maltose syrup seems to be disturbed. In particular, this is indicated by the highest degree of proline utilization, despite the fact that the wort still has amino acids that are considered to be better assimilated by yeasts.

### 3.6. The Effect of Amino Acid Profile in Worts on the Synthesis of Fermentation Byproducts by Yeast

The different amino acid profiles of worts significantly affected beer’s flavor bouquet. Yeasts, by deamination, decarboxylation, and reduction of amino acids, synthesize higher alcohols, of which to a large extent are created, among others, esters [35]. Literature data show that individual amino acids can be converted into specific higher alcohols. For example, 2- and 3- methylbutanol is formed from isoleucine and leucine, isobutanol is synthesized from valine, while phenylethanol is produced from phenylalanine [35,36]. These relationships have also been noted in the conducted research. In beer obtained with 30% addition of amaranth, where a greater content of valine was noted, an increase in isobutanol content was found (Table 4). In contrast, the lower concentration of the 2- and 3-methylbutanol and phenylethanol was noted in the beer with a reduced amount of leucine, isoleucine, and phenylalanine. In the case of wort obtained with 30% quinoa, analogous results were obtained [14]. According to our previous results [14] and the findings of this study (Table 4), lower FAN content in worts obtained with 30% quinoa or amaranth also contributes to the synthesis of smaller amounts of higher alcohols. In turn, during fermentation of the wort obtained with a 30% maltose syrup, in which the concentration of individual amino acids was the lowest, yeasts synthesized the smallest amount of higher alcohols (53.9 mg/L).

Compared to the esters content in beer produced exclusively from barley malt (8.85 mg/L) [14], beers obtained with the use of 30% quinoa (7.89 mg/L) [14], amaranth, or maltose syrup were characterized by a lower total content of determined esters (Table 4). Esters are products in the way of enzymatic condensation between higher alcohols and organic acids. Thus, the reduced synthesis of higher alcohols by yeast also had an impact on the reduced content of esters in beers produced with unmalted raw materials. The lowest concentration of fatty acids was noted in the wort obtained with 30% maltose syrup, but the content of synthesized esters in beer with this raw material is the lowest. Therefore, it can be assumed that in this case, the content of higher alcohols, and at the same time FAN, had the greatest influence on the concentration of esters in beer.

## 4. Conclusions

The proper propagation and metabolism of yeasts, as well as resulting fermentation, requires an optimal concentration of nutrients, macro- and microelements in the wort. It is important that the fermentation medium contains a sufficient amount of unsaturated long-chain fatty acids and low-molecular nitrogen compounds, mainly amino acids belonging to the group A. Although the replacement of a part of malt with quinoa or amaranth resulted in a decrease in FAN content, analysis of the amino acid profiles showed that in the case of worts with the pseudocereals, the share of amino acids assimilated by yeast was increased. Therefore, the use of unmalted pseudocereals in the production of wort positively influenced the amino acid profiles, which resulted in a greater degree of assimilation of these compounds by yeasts. Wort obtained with a 30% of maltose syrup was characterized by the lowest content of low molecular weight nitrogen compounds and the least favorable amino acid profile. Consequently, this resulted in the lowest degree of utilization of these compounds by yeasts. On the other hand, in worts obtained with 30% of pseudocereals, a higher content of fatty acids, including long-chain and unsaturated, was determined. These compounds were the most used by yeasts during fermentation. In addition, a higher concentration of fatty acids, such as oleic, palmitic, linoleic, or α-linolenic acid was noted in worts obtained with the addition of pseudocereals.

Differences in the profiles of amino acids and fatty acids in worts significantly influenced the concentration of flavors synthesized by yeast during fermentation. The reduced content of low-molecular nitrogen compounds and the increased amount of fatty acids in worts obtained with the participation of pseudocereals resulted in the limited synthesis of higher alcohols and esters.

The research showed that replacing part of the malt with pseudocereals during the production of wort, in particular with quinoa, significantly affects the enrichment of the wort with compounds necessary for brewer’s yeasts. Consequently, this can have a positive effect on their activity during the fermentation process.

## Figures and Tables

**Table 1:** The fatty acid profile in wort and beer produced only from barley malt and with addition of unmalted pseudocereals or syrup.

Fatty Acid (mg/L)	100% Barley Malt		30% Amaranth Flakes		30% Quinoa Flakes		30% Maltose Syrup	
	Wort	Beer	Wort	Beer	Wort	Beer	Wort	Beer
Caproic (C6:0)	0.20 ± 0.01 ^a^	0.15 ± 0.01 ^1^	0.22 ± 0.02 ^b^	0.18 ± 0.01 ^2^	0.24 ± 0.02 ^b^	0.16 ± 0.02 ^1^	0.17 ± 0.01 ^c^	0.12 ± 0.01 ^4^
Caprylic (C8:0)	0.24 ± 0.02 ^a^	0.20 ± 0.01 ^1^	0.30 ± 0.01 ^b^	0.28 ± 0.01 ^2^	0.26 ± 0.02 ^a^	0.24 ± 0.02 ^3^	0.17 ± 0.01 ^c^	0.16 ± 0.01 ^4^
Capric (C10:0)	0.22 ± 0.02 ^a^	0.20 ± 0.00 ^1^	0.25 ± 0.01 ^b^	0.23 ± 0.00 ^2^	0.23 ± 0.01 ^a^	0.23 ± 0.00 ^2^	0.15 ± 0.01 ^c^	0.14 ± 0.01 ^3^
Lauric (C12:0)	0.22 ± 0.01 ^a^	0.20 ± 0.00 ^1^	0.26 ± 0.00 ^b^	0.22 ± 0.01 ^2^	0.26 ± 0.01 ^c^	0.20 ± 0.00 ^1^	0.16 ± 0.01 ^d^	0.13 ± 0.01 ^3^
Myristic (C14:0)	0.23 ± 0.01 ^a^	0.10 ± 0.00 ^1^	0.15 ± 0.02 ^b^	0.10 ± 0.00 ^1^	0.15 ± 0.03 ^b^	0.10 ± 0.00 ^1^	0.20 ± 0.01 ^c^	0.16 ± 0.01 ^2^
Palmitic (C16:0)	0.50 ± 0.02 ^a^	0.20 ± 0.00 ^1^	0.77 ± 0.02 ^b^	0.24 ± 0.01 ^2^	0.71 ± 0.03 ^c^	0.19 ± 0.02 ^3^	0.45 ± 0.03 ^d^	0.15 ± 0.01 ^4^
Margaric (C17:0)	0.15 ± 0.01 ^a^	0.08 ± 0.00 ^1^	0.27 ± 0.01 ^b^	0.08 ±0.00 ^1^	0.25 ± 0.01 ^b^	0.10 ± 0.02 ^2^	0.15 ± 0.01 ^a^	0.07 ± 0.01 ^3^
Stearic (C18:0)	0.67 ± 0.01 ^a^	0.39 ± 0.00 ^1^	0.81 ± 0.02 ^b^	0.48 ± 0.02 ^2^	0.75 ± 0.06 ^c^	0.43 ± 0.01 ^3^	0.32 ± 0.02 ^d^	0.28 ± 0.01 ^4^
Oleic cis (C18:1)	1.04 ± 0.04 ^a^	0.15 ± 0.00 ^1^	1.46 ± 0.01 ^b^	0.20 ± 0.01 ^2^	1.34 ± 0.04 ^c^	0.10 ± 0.01 ^3^	0.65 ± 0.01 ^d^	0.20 ± 0.01 ^2^
Linoleic cis (C18:2)	0.21 ± 0.02 ^a^	0.07 ± 0.00 ^1^	0.30 ± 0.01 ^b^	0.11 ± 0.02 ^2^	0.37 ± 0.01 ^c^	0.14 ± 0.01 ^3^	0.17 ± 0.01 ^d^	0.08 ± 0.00 ^4^
α-Linolenic (C18:3 *n*-3)	0.36 ± 0.04 ^a^	0.05 ± 0.00 ^1^	0.55 ± 0.01 ^b^	0.07 ± 0.01 ^2^	0.45 ± 0.00 ^c^	0.05 ± 0.00 ^1^	0.15 ± 0.01 ^d^	0.03 ± 0.01 ^3^
γ-Linolenic (C18:3 *n*-6)	0.07 ± 0.00 ^a^	0.03 ± 0.00 ^1^	0.19 ± 0.01 ^b^	0.06 ± 0.01 ^2^	0.16 ± 0.00 ^c^	0.03 ± 0.00 ^1^	0.04 ± 0.00 ^d^	0.02 ± 0.01 ^3^
Arachidic (C20:0)	0.09 ± 0.00 ^a^	0.05 ± 0.00 ^1^	0.13 ± 0.02 ^b^	0.09 ± 0.01 ^2^	0.12 ± 0.00 ^c^	0.08 ± 0.01 ^2^	0.06 ± 0.00 ^f^	0.03 ± 0.02 ^3^
Arachidonic (C20:4)	0.20 ± 0.03 ^a^	0.17 ± 0.00 ^1^	0.20 ± 0.03 ^a^	0.15 ± 0.02 ^2^	0.22 ± 0.02 ^a^	0.18 ± 0.00 ^3^	0.09 ± 0.00 ^f^	0.07 ± 0.01 ^4^
Total (mg/L)	4.40 ± 0.02 ^a^	2.04 ± 0.01 ^1^	5.86 ± 0.01 ^b^	2.46 ± 0.02 ^2^	5.51 ± 0.02 ^c^	2.23 ± 0.01 ^3^	2.93 ± 0.01 ^d^	1.64 ± 0.01 ^4^

Results expressed as mean values ± SE (*n* = 3); values with superscript different letters (worts) or number (beers) in the same line are significantly different (*p* < 0.05).

**Table 2:** The concentration of the free amino nitrogen in worts and beers.

Type of Wort and Beer	FAN (mg/L)		Assimilation (%)
	Wort	Beer	
100% Barley malt	240.7 ± 2.7	78.2 ± 3.2 ^a^	68
30% Amaranth flakes	208.4 ± 6.2 ^1^	56.5 ± 5.2 ^a,1^	73
30% Quinoa flakes	198.9 ± 4.8 ^1^	46.2 ± 2.6 ^a,1^	77
30% Maltose syrup	134.1 ± 3.9 ^1^	72.2 ± 5.3 ^a,2^	46

FAN—Free amino nitrogen. Lowercase letter—statistical analysis between wort and beer—^a^: *p* < 0.0001. Lowercase numbers—statistical analysis within wort or beer where control was 100% barley malt—^1^: *p* < 0.0001; ^2^—0.00001 < *p* < 0.05.

**Table 3:** The content of the amino acid in wort and beer produced with the addition of unmalted raw materials.

Amino Acid (mg/L)		100% Barley Malt		30% Amaranth Flakes		30% Quinoa Flakes		30% Maltose Syrup	
		Wort	Beer	Wort	Beer	Wort	Beer	Wort	Beer
Group A	Lysine	32.9 ± 2.4 ^a^	0.00 ^1^	40.8 ± 2.7 ^b^	0.00 ^1^	44.6 ± 1.5 ^c^	0.00 ^1^	19.7 ± 2.1 ^d^	0.00 ^1^
	Asparagine	35.3 ± 2.2 ^a^	3.2 ± 0.2 ^1^	38.9 ± 6.1 ^b^	4.8 ± 0.4 ^2^	36.4 ± 2.3 ^a^	3.1 ± 0.2 ^1^	21.2 ± 2.3 ^d^	0.00 ^3^
	Glutamine	39.7 ± 2.3 ^a^	4.5 ± 0.1 ^1^	42.6 ± 1.1 ^b^	3.4 ± 0.6 ^2^	46.5 ± 3.4 ^c^	5.1 ± 0.9 ^3^	25.8 ± 1.5 ^d^	0.00 ^3^
	Serine	25.0 ± 2.1 ^a^	0.00 ^1^	37.8 ± 2.2 ^b^	3.1 ± 0.7 ^2^	36.6 ± 3.1 ^b^	3.0 ± 0.6 ^2^	16.2 ± 2.2 ^d^	0.00 ^3^
	Arginine	61.8 ± 7.2 ^a^	0.00 ^1^	62.1 ± 6.9 ^a^	4.32 ± 0.9 ^2^	62.2 ± 6.7 ^a^	0.00 ^1^	46.4 ± 5.6 ^d^	3.2 ± 0.3 ^3^
	Threonine	39.1 ± 4.2 ^a^	4.2 ± 0.4 ^1^	20.9 ± 1.5 ^b^	3.0 ± 0.6 ^2^	45.2 ± 1.4 ^c^	4.4 ± 0.2 ^1^	19.6 ± 2.5 ^d^	0.00 ^3^
Group B	Histidine	15.8 ± 1.5 ^a^	8.2 ± 0.6 ^1^	19.1 ± 2.0 ^b^	4.0 ± 0.4 ^2^	18.3 ± 2.6 ^b^	10.2 ± 2.3 ^3^	9.5 ± 2.4 ^c^	4.3 ± 0.3 ^2^
	Valine	47.0 ± 3.2 ^a^	15.2 ± 2.2 ^1^	49.4 ± 3.4 ^a^	14.2 ± 2.5 ^1^	34.2 ± 2.9 ^b^	16.5 ± 2.7 ^1^	32.9 ± 3.2 ^b^	25.5 ± 3.5 ^2^
	Methionine	29.0 ± 1.4 ^a^	0.00 ^1^	32.2 ± 4.1 ^b^	0.00 ^1^	35.3 ± 2.0 ^c^	0.00 ^1^	17.4 ± 2.7 ^d^	0.00 ^1^
	Isoleucine	40.1 ± 1.1 ^a^	10.3 ± 0.9 ^1^	35.7 ± 3.2 ^b^	12.9 ± 1.8 ^2^	41.3 ± 0.5 ^a^	5.5 ± 0.8 ^3^	26.0 ± 4.3 ^c^	6.2 ± 0.6 ^4^
	Leucine	90.7 ± 2.3 ^a^	22.7 ± 2.2 ^1^	60.8 ± 4.5 ^b^	17.0 ± 2.1 ^2^	100.5 ± 4.8 ^c^	10.4 ± 0.9 ^3^	63.5 ± 3.6 ^b^	35.2 ± 3.7 ^4^
Group C	Alanine	91.0 ± 8.7 ^a^	65.2 ± 6.7 ^1^	95.9 ± 1.2 ^b^	65.0 ± 5.4 ^1^	50.4 ±4.1 ^c^	42.1 ± 6.9 ^2^	72.8 ± 6.5 ^d^	30.2 ± 6.2 ^3^
	Glycine	17.6 ± 2.3 ^a^	12.2 ± 2.7 ^1^	5.9 ± 1.1 ^b^	5.0 ± 1.4 ^2^	17.5 ± 2.3 ^a^	16.2 ± 1.7 ^3^	12.3 ± 2.2 ^d^	5.2 ± 1.1 ^2^
	Tyrosine	76.3 ± 6.7 ^a^	50.1 ± 8.2 ^1^	28.1 ± 2.9 ^b^	26.0 ± 3.2 ^2^	55.2 ± 5.2 ^c^	50.0 ± 6.2 ^1^	57.2 ± 4.7 ^c^	42.4 ± 3.6 ^3^
	Phenylalanine	71.7 ± 3.3 ^a^	22.2 ± 3.1 ^1^	39.9 ± 3.2 ^b^	28.1 ± 2.7 ^2^	20.1 ± 1.9 ^c^	14.2 ± 2.5 ^3^	50.2 ± 2.6 ^d^	30.3 ± 2.8 ^2^
Group D	Proline	290.2 ± 11.3 ^a^	285.7 ± 2.7 ^1^	205.4 ± 3.4 ^b^	203.2 ± 3.9 ^2^	190.1 ± 0.9 ^c^	189.8 ± 1.1 ^3^	226.3 ± 2.5 ^d^	218.4 ± 3.4 ^4^
Total (mg/L)		1003.2 ± 3.8 ^a^	504 ± 1.9 ^1^	816 ± 3.2 ^b^	394 ± 1.7 ^2^	834 ± 5.1 ^c^	370 ± 1.6 ^3^	717 ± 3.2 ^d^	400 ± 1.8 ^4^

Results expressed as mean values ± SE (*n* = 3); values with superscript different letters (worts) or number (beers) in the same line are significantly different (*p* < 0.05).

**Table 4:** The concentration of the higher alcohols and esters in beer.

Higher Alcohol (mg/L)	Type of Beer	
	30% Amaranth Flakes	30% Maltose Syrup
1-proponaol	10.3 ± 0.2	12.6 ± 0.3
isobutanol	17.4 ± 0.5	11.3 ± 0.3
2- and 3- methylbutanol	19.1 ± 0.2	18.2 ± 0.2
2-phenylethanol	13.3 ± 0.9	11.8 ± 1.1
Total higher alcohols (mg/L)	60.1 ± 1.8	53.9 ± 1.9
Total esters (mg/L)	6.93 ± 0.14	6.16 ± 0.10
